# Expulsion of Entire Ova

**Published:** 1846-04

**Authors:** 


*Expulsion of Entire Ova.*—Dr. Mackness relates a case of twins, both of which were expelled separately, enclosed in the membranes, and when the latter were opened appeared to be still-born. By assiduously endeavouring to excite the function of respiration by inflating the lungs, dashing cold water over their bodies, wiping them dry, and having their skins well rubbed, both infants showed signs of animation, and on being exposed to the warmth of a fire, cried lustily. *Ibid.*

